# 3D printed microneedles: revamping transdermal drug delivery systems

**DOI:** 10.1007/s13346-024-01679-7

**Published:** 2024-08-05

**Authors:** Ashlesh Prabhu, Vishal Baliga, Raghavendra Shenoy, Akanksha D. Dessai, Usha Y. Nayak

**Affiliations:** Department of Pharmaceutics, Manipal College of Pharmaceutical Sciences, Manipal Academy of Higher Education, Manipal, 576104 Karnataka India

**Keywords:** 3D printing, Microneedles, Transdermal drug delivery systems, Fused deposition modelling, Stereolithography

## Abstract

One of the advancements of the transdermal drug delivery system (TDDS) is the development of microneedles (MNs). These micron-sized needles are used for delivering various types of drugs to address the disadvantage of other transdermal techniques as well as oral drug delivery systems. MNs have high patient acceptance due to self-administration with minimally invasive and pain compared to the parenteral drug delivery. Over the years, various methods have been adopted to evolve the MNs and make them more cost-effective, accurate, and suitable for multiple applications. One such method is the 3D printing of MNs. The development of MN platforms using 3D printing has been made possible by improved features like precision, printing resolution, and the feasibility of using low-cost raw materials. In this review, we have tried to explain various types of MNs, fabrication methods, materials used in the formulation of MNs, and the recent applications that utilize 3D-printed MNs.

## Graphical abstract

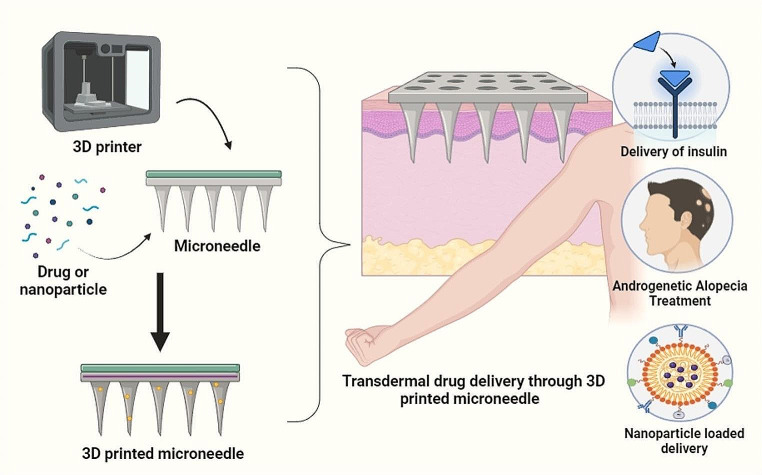

## Introduction

Transdermal drug delivery (TDD) involves the delivery of the drug through the skin, and it offers an attractive, non-invasive systemic route other than parenteral and oral drug delivery systems with painless drug delivery, no first-pass metabolism, improved patient compliance, self-administration, and minimally invasive drug therapy. Additionally, this route enhances the bioavailability of the drugs and, hence, their local and systemic treatment. Researchers delved into TDD and dosage forms like transdermal and iontophoretic patches that have been clinically accepted and marketed [1–3]. To understand how the drug penetrates the skin, it is essential to discuss the layers of the skin. Human skin contains a dermis, epidermis, and hypodermis, out of which the epidermis contains various layers such as stratum granulosum, stratum corneum, stratum lucidum, stratum spinosum, and stratum basale. The stratum corneum contains dead keratinized cells and corneocytes and is highly hydrophobic, preventing the passage of most drugs through the skin [4]. It acts as a barrier to the skin for protection against various agents. Therefore, smaller particles with low molecular weight can be easily penetrated; however, larger molecules require special techniques [5].

**Fig. 1:**
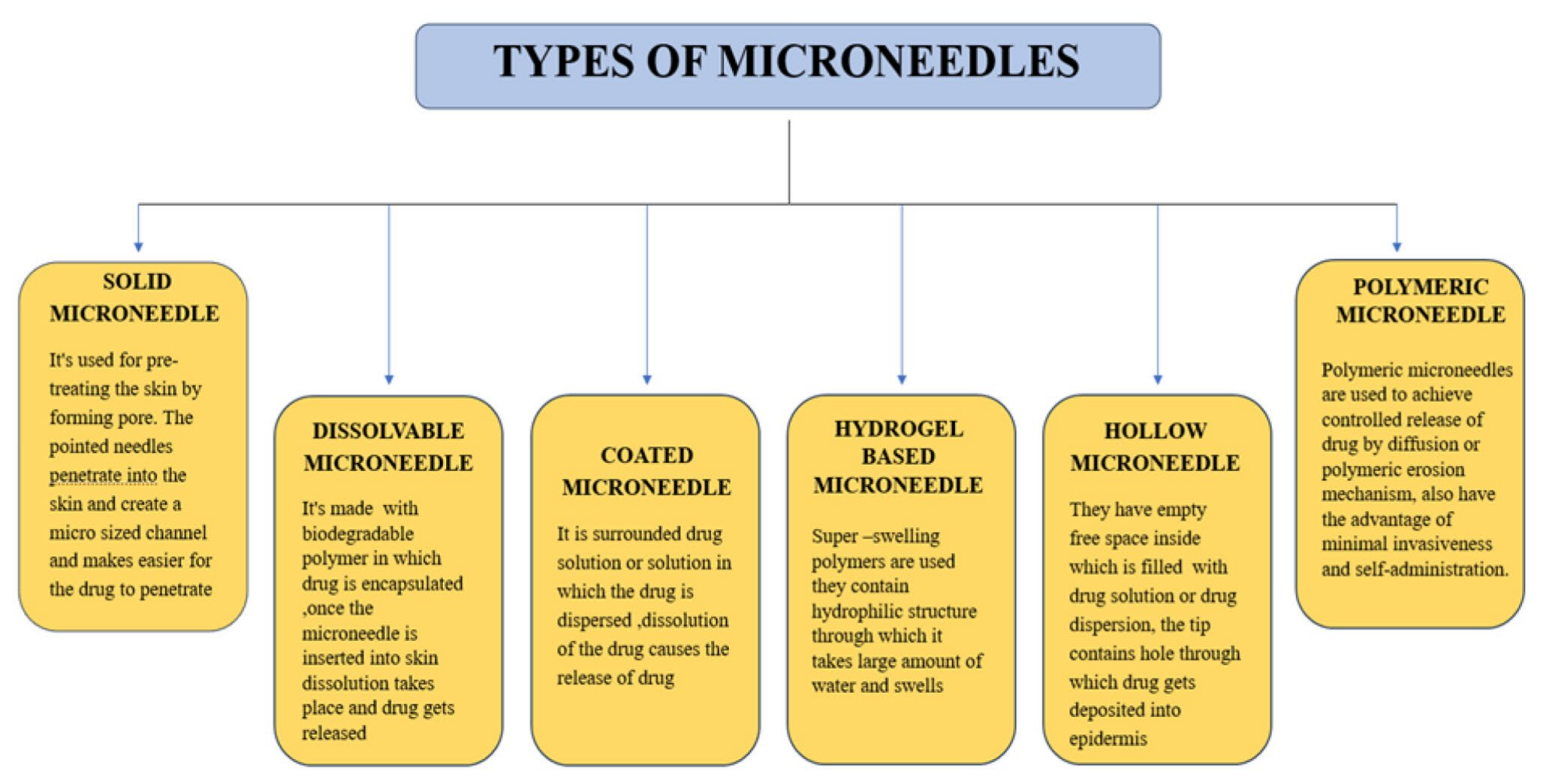
Classification of microneedles

Microneedles (MNs) are micron-sized structures that help to penetrate the drug through the stratum corneum. MNs comprise diverse components, such as ceramics, various polymers, and metals, intended for the epidermal and intradermal delivery of vaccines and bioactive molecules [6]. The primary mode of MN drug delivery involves establishing a micron-sized channel by damaging the skin barrier [7]. Although one of the critical barriers to the delivery of the drugs through MN is the outermost layer of skin, which is the stratum corneum, to improve transdermal medication delivery, a variety of techniques have been employed, involving sonophoresis, iontophoresis, and electroporation technologies. MNs have been used to increase the concentration of the drug at the delivery site [8]. Also, in combination with chemicals, the release of the drug in the systemic circulation can be enhanced [9]. Studies are being conducted on transdermal patches created with these techniques to address the barrier problem of stratum corneum [10]. Sonophoresis is the process of delivering medication via the skin using ultrasonography technology. It works primarily by creating holes in the skin’s layers through oscillation, pressure gradients, and local heating, which improves medication delivery. The thermal effect of ultrasonography has a potential effect on the drug diffusion coefficient [11]. Iontophoresis is a technique that enhances medication delivery by providing a low-voltage current to the skin layer, during which charged drug particles diffuse through the skin [12]. Meanwhile, the short-term application of high voltage current creates cavities in the skin layer, improving medication delivery. This process is called electroporation [13]. MNs are widely accepted by patients and clinicians. There are various types of MNs: solid MN, dissolvable MN, coated MN, hydrogel-based MN, hollow MN, and polymeric MN. The classification of MNs is indicated in Fig. 1 [14]. The base may be chosen depending on the need for delivery during fabrication.

In 1979, the US FDA authorized the first transdermal patch containing scopolamine to treat motion sickness. Recently, MNs have garnered much interest as a novel TDD method because of their distinctive advantages, which include low invasiveness, self-administration, and painless delivery because of their micron-sized structure [15].

**Fig. 2:**
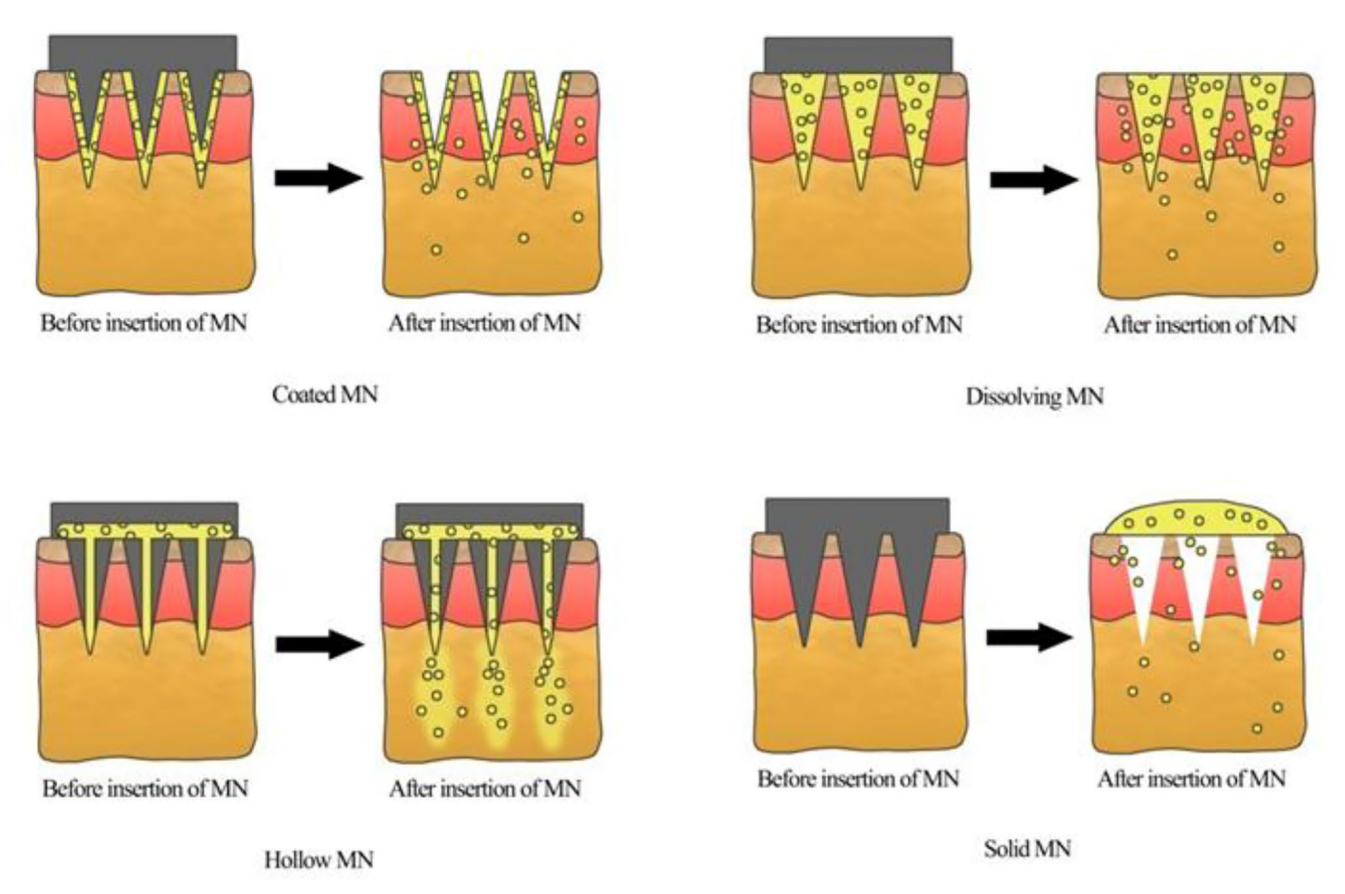
Mechanism of drug release in different types of MNs

MNs have been able to deliver various types of agents like small drug molecules, macromolecules (peptides, proteins, vaccines, nucleic acids, etc.), and nanoparticles to pass through the sub-cutaneous layer and reach the systemic circulation directly [16]. The delivery mechanism of the MNs is shown in Fig. 2. Conventional methods of fabricating MNs involve wet and dry etching methods, lithography, molding, and laser cutting, through which various types of MNs can be manufactured. Still, the drawbacks of these conventional methods include cost-effectiveness and complexity; some require manual operation involving several manufacturing steps, a complex process [17].

Additive manufacturing, or 3D printing, is a contemporary method in which objects are constructed layer by layer by imitating computer-aided design (CAD) models. This technique can produce complex structures, higher precision, and high drug loading capacity, which makes it extremely capable of delivering tailored medicines and medical devices that effectively overcome the limits associated with conventional dosage forms [18]. The 3D printing technique was developed and implemented in the pharmaceutical field in the late 90s, which was done by inkjet printing using a liquid binder solution into a powder mass [19]. 3D printing technology empowers the customization of dosage forms according to individuals’ needs by creating specific dosage forms, altering dosages, or differing release profiles. The primary population requiring dose flexibility is the pediatric group, wherein the therapeutic dosage is adjusted based on children’s age and body weight [20]. Different methods have been used for 3D printing, such as powder bed fusion, binder jetting, and material extrusion, for various dosage form preparations [21]. This review discusses various types of MNs, fabrication methods of 3D printed MNs, materials used in the formulation of MNs, and the applications that utilize 3D-printed MNs.

## Need of 3D printing for MN

Many old techniques of MN fabrication have disadvantages, such as the need for skilled workers, economic viability, and heavy labour. Advanced techniques with minimal disadvantages, such as 3D printing, are needed to address this drawback. 3D printing technology can potentially evolve MN-based drug delivery systems in the future. The need for inexpensive, compact, reliable, accurate, and readily accessible MNs with a variety of shapes and types, bio-signal acquisition features, sample extraction from the body, and diagnostic testing of these features are required, which can be obtained from the 3D printing technique. 3D printing can effectively create, update, and manufacture MNs with appropriate size characteristics and thus reduce the time required for design and prototyping by eliminating intermediary processes, which are prerequisites for contract manufacturing firms. The slow printing, resolution-related limitations, material limitations, and biocompatibility issues are all significant disadvantages to 3D printing, and the actual technique of 3D printing is slower than conventional manufacturing methods like injection molding. Furthermore, despite the widespread interest in 3D printing, obtaining higher resolution remains a challenge [21, 22]. Compared to conventional methods MN production by 3D printing is cost-effective as the material wastage is reduced. 3D printing allows for more efficient use of materials by only depositing the required amount in the exact locations needed. This minimizes material waste compared to subtractive manufacturing processes or traditional tablet compression. 3D printing allows for design flexibility as the layer-by-layer 3D printing process enables the fabrication of complex geometries and customized dosage forms that would be difficult or impossible to produce using conventional manufacturing. This design flexibility can lead to improved drug delivery and reduced costs. The third significant advantage of 3D printing over traditional drug delivery is the manufacture of personalized medicine. It enables the production of patient-specific dosage forms tailored to individual needs. This can eliminate the need for costly inventory of multiple standard tablet strengths and formulations, reducing overall costs [23, 24].

## Methods of manufacturing 3D printed MNs

### Fused deposition modelling (FDM)

FDM, also known as fused filament fabrication (FFF), is a recently developed method for printing MN arrays with specific needle density, length, and form requirements. It is recognized as one of the most affordable and accessible 3D printing systems. The thermoplastic materials serving as starting materials that have the potential to change their shape when introduced “as filaments” into the FDM 3D printer. These smoothed thermoplastic filaments are ejected from the printer’s head and can move across X and Y planes. The product is formed by melting thermoplastic filaments in several layers, extruding them from the printer’s head, and then solidifying layer by layer when they reach the underlying bed. Once the primary layer solidifies, another layer of the molten filament can be deposited over it [25, 26].

**Fig. 3:**
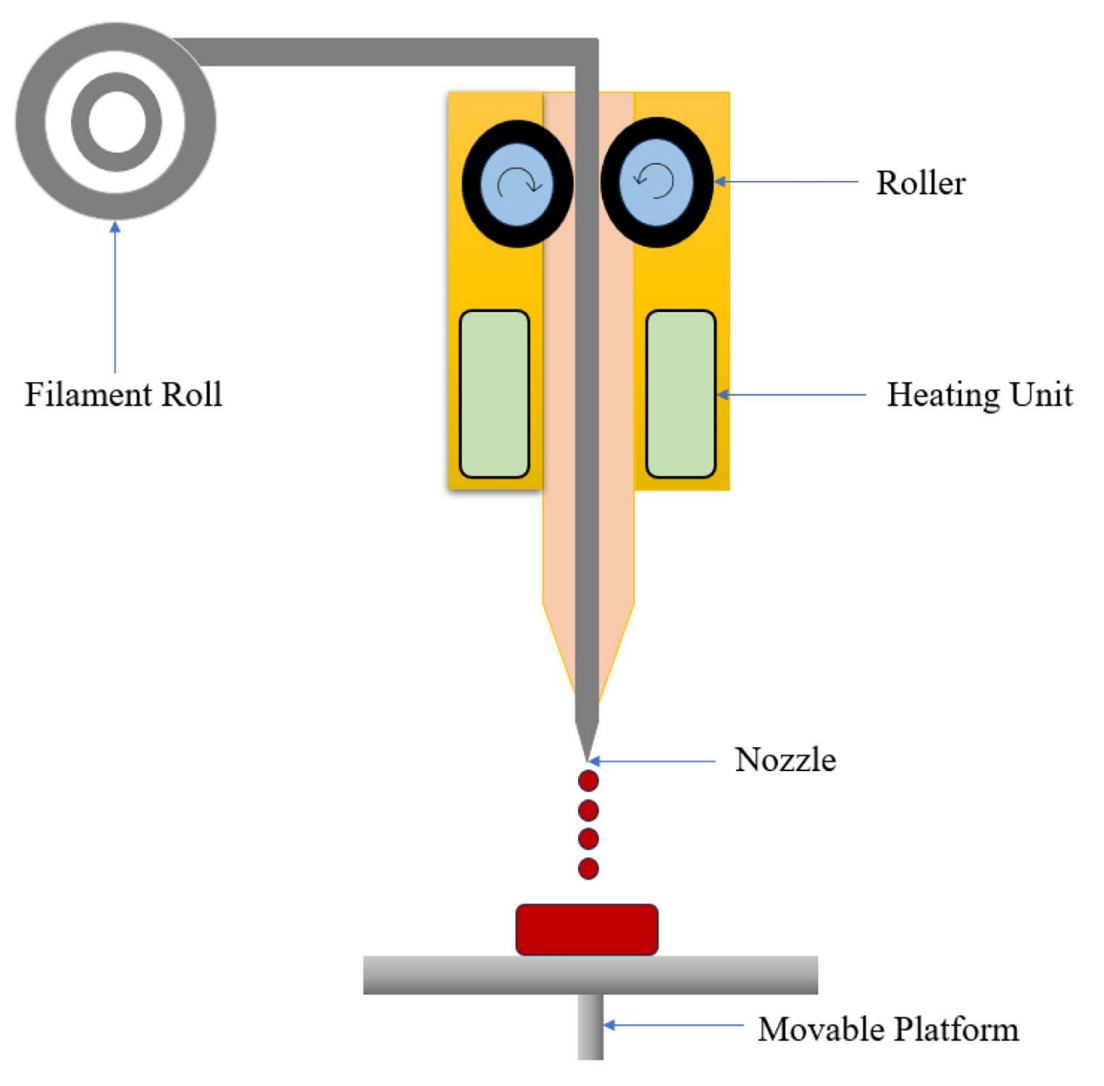
Schematic diagram of working of FDM

Prototype polymeric MN arrays were investigated by several researchers using FDM 3D printing. Luzuriaga and coworkers used the FDM approach in combination with a chemical etching post-methods strategy to create biodegradable and biocompatible MNs with adequate accuracy. Based on their findings, it was possible to produce 3D-printed MNs with a suitable tip size using the chemical etching approach. The production of MNs, was found to be an essential tool for achieving an appropriate balance between more efficiency and reduced cost [27]. FDM is essentially used in pharmaceutical dosage types, such as MNs and tablets. Still, one of the critical challenges of FDM is low resolution, low processing speed, and greater precision, which is a significant downside in the manufacture of MNs. Its illustration is shown in Fig. 3 [17].

### Two-photon polymerization (2PP)

**Fig. 4:**
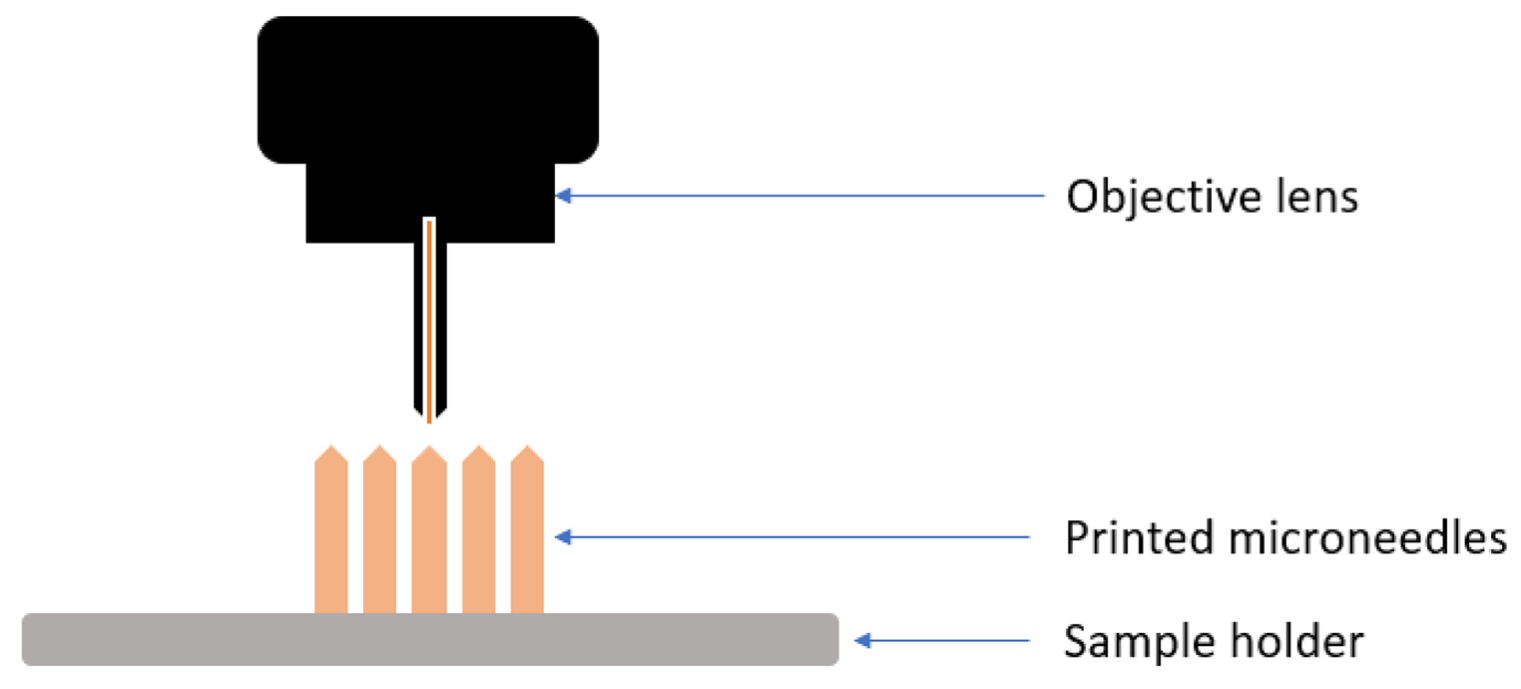
Schematic diagram of working of 2PP

The discovery of the 2PP allowed the production of intricate micro and nanoscale structures, whose structure is shown in Fig. 4. Using this technique, photosensitive resins are selectively polymerized by applying ultrashort laser pulses from a near-infrared femtosecond laser source. The level of excitation created by absorbing two photons is equivalent to one photon with more incredible energy due to irregular energy distribution occurring at the center of the laser focal point and outside the focused laser point. When photo-initiator molecules in the resin absorb energy and reach a threshold, they initiate the polymerization process in those locations, referred to as polymerization voxels. This is the working principle of the instrument. It is considered a very accurate 3D printing method for developing several sophisticated structures [28].

Cordeiro et al. (2020) employed 2PP, a 3D printing technology, to create MN array templates with changing needle height, shape, form (conical, pyramidal, cross-shaped, and pedestal), and base width. The study aimed to develop a range of MN templates in arrays with a variable needle shape, size, and geometries and compare the performance of these MN arrays. Subsequently, these templates enabled us to produce reproducible, reusable MN array moulds, which could potentially be applied to produce hydrogel-generating MN arrays that may be used as drug delivery systems. The potential of the MN arrays to transport drugs such as cabotegravir sodium and ibuprofen sodium to viable layers of the skin was evaluated ex vivo and in vitro for controlled release and absorption [28].

### Stereo lithography appearance (SLA)

**Fig. 5:**
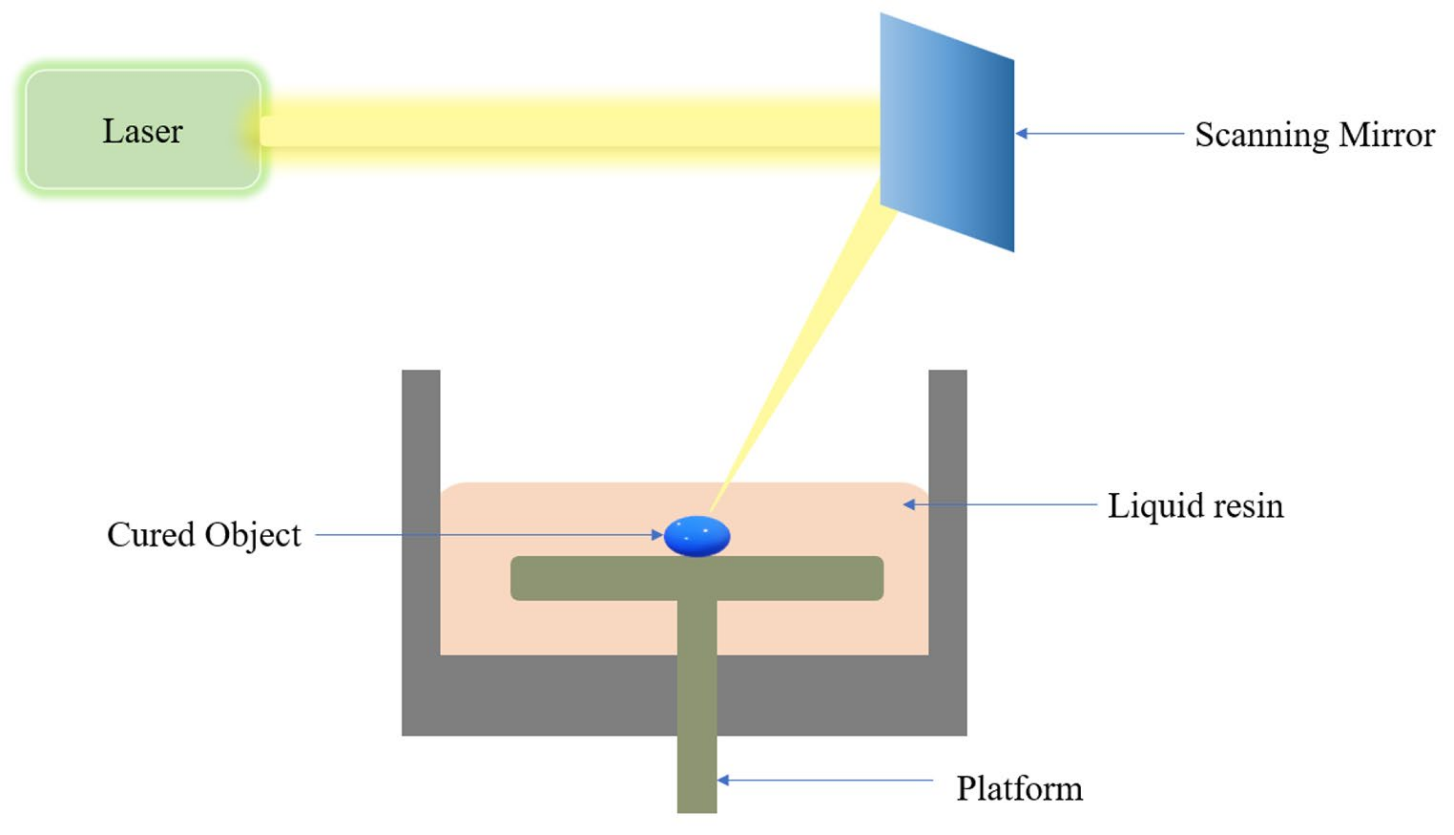
Schematic diagram of working of SLA

Stereolithography is the most well-known and extensively used 3D printing technology among all the technologies used in 3D printing. The illustration of the instrument is shown in Fig. 5. In 1986, Charles Hull became the first person to create and patent SLA-based 3D printing technology, a Vat photopolymerization process (Patent no. US6027324A). The terms “photolithography” and “stereo (solid) " refer to “writing with light.” Components employed in SLA methods solidify, remarkably, UV (Ultraviolet) light (Photopolymerizable) upon exposure to light. Employing a spatially controlled laser so that the light is exposed selectively, the size and shape of the object can be altered [29]. The technique involves the use of a computer-controlled UV laser beam for photopolymerization. The laser beam is directed towards liquid resin and is cured layer by layer until a 3D object is formed. Applying a laser beam simplifies and facilitates the production of highly accurate objects with error-free surface finish. The amount of energy supplied by the laser is essential in SLA printing. It is majorly affected by the strength of the light source, scanning speed, time of exposure, and amount of polymer and photoinitiator [30]. SLA techniques can be used to create several types of MNs, such as solid MNs and hollow MNs.

Yeung and colleagues created a hollow MN with a microfluidic system for effective transdermal medication administration. Effective integration of the microfluidic systems with printed MNs enables cheaper costs, faster speeds, and higher throughput than previously published methods, as reported by the authors. The entire system might also allow the homogenous mixing of many fluids at various flow rates, followed by a uniform distribution of uniformly blended solutions. According to their findings, the innovative use of 3D-printed MNs with a microfluidics system offers a possible substitute for rapid and effective transdermal medication administration [31]. Uddin and colleagues described a unique cross-shape SLA-printed MN array by utilizing cisplatin as the model drug and coating the surface of the printed MNs using an inkjet technique. Their test findings showed that the unique cross-shaped MNs had a remarkable capability for skin penetration. The in vivo assessment also showed adequate cisplatin penetration, strong anticancer efficacy, and tumor inhibition [32].

### Digital light processing (DLP)

**Fig. 6:**
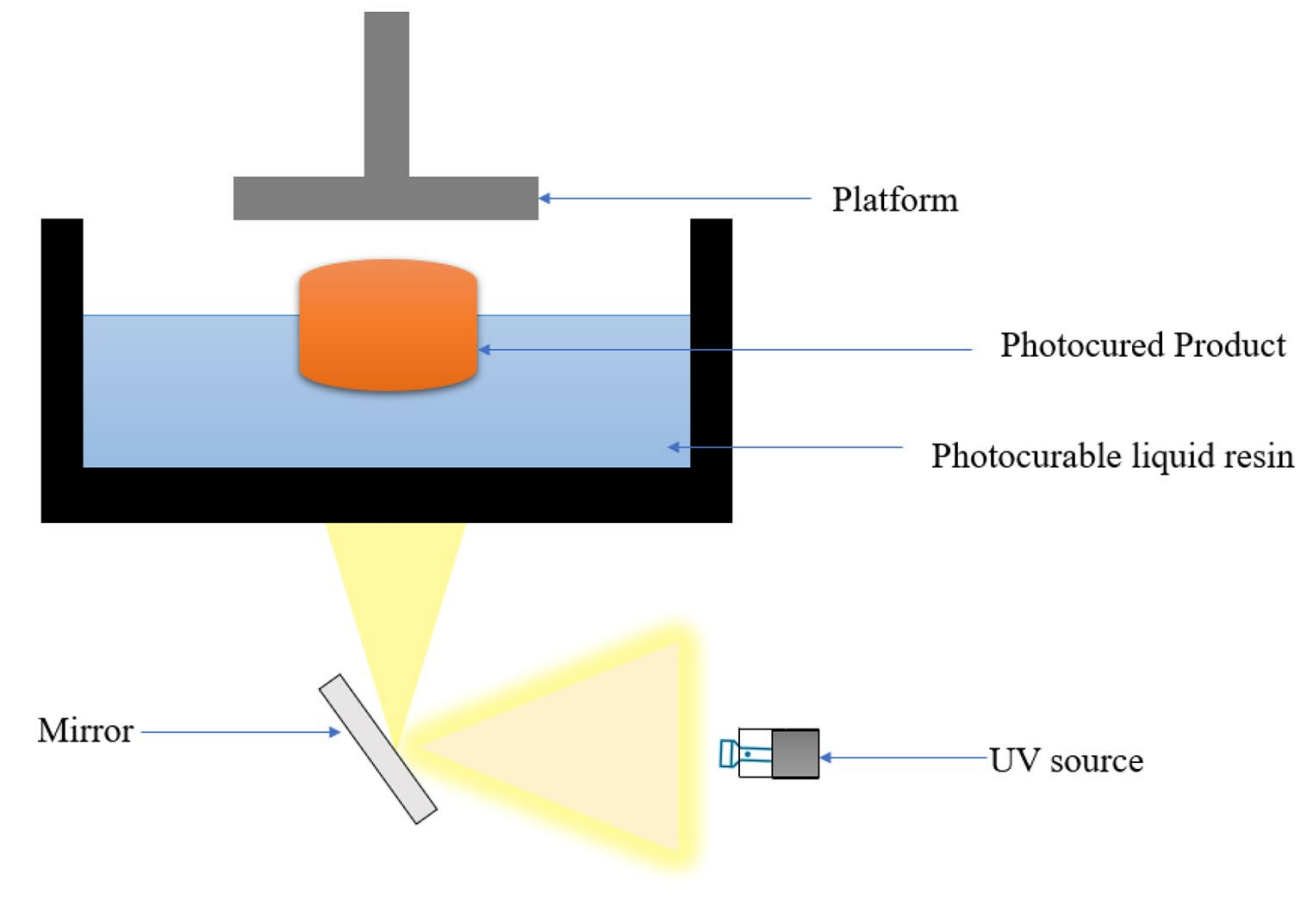
Schematic diagram of working of DLP

DLP is another type of vat polymerization technique that is comparable to SLA. A DLP system comprises a digital micromirror and a digital projector to emit light of minute pixels in rectangular cube-shaped form, depicting an entire cross-sectional layer of the object on photopolymerizable resin as shown in Fig. 6 [33]. A 3D digital model is first sliced into thin layers using specialized software. The principle works as follows: the DLP printer has a Vat filled with a liquid photopolymer resin. A high-resolution digital light projector shines UV light onto the resin’s surface, projecting the current layer’s shape. The projected UV light triggers a photopolymerization reaction, hardening the resin in the illuminated areas and forming a solid layer. The build platform then moves down by a small distance, exposing a new layer of liquid resin. The projector flashes the UV light again, curing the new layer and adhering it to the previous one. This process is repeated, building the 3D object layer-by-layer until the entire part is complete. When light is directed onto the photopolymerizable resin, the whole cross-sectional area of the object solidifies. Thus, the object generated will have rough surfaces due to the presence of rectangular cubes known as voxels. DLP technologies print objects faster than SLA because they polymerize an entire cross-section instantly. However, in SLA and DLP systems, the formed layer separates from the base of the Vat, extending the production time [34]. Lim and colleagues addressed the problem of ineffective drug delivery across the undulating skin surface by creating a customized dual-function MN splint to treat the trigger finger. According to the assessment findings, the DLP-printed MN splint has the appropriate physical features, including qualifying mechanical properties and sufficient skin penetration capacity [35].

DLP can also create a suitable mould for the MNs using the DLP 3D printing technology. El Sayyed and colleagues cost-effectively developed MNs. The initial master mould was created using a desktop DLP 3D printer. To improve the MNs’ penetration ability into the skin, they chose a design for the MNs that was ‘tanto blade’ inspired. Tanto refers to a classic Japanese Samurai sword with a unique design called tanto. It features two bevels: one at the front that is shorter and more vertical and one that continues to the hilt and is long and straight, with a beveled tip that enhances penetration. Tanto blade designs are stable and sharp, making putting MNs into the skin easier. Also, they are less bulky, so moving or otherwise disrupting the skin barrier is impossible. Tanto blade-inspired MNs demonstrated effective skin penetration without pre-mature fracture, as the fracture force was more significant than the insertion force [36].

### Continuous liquid interface production (CLIP)

**Fig. 7:**
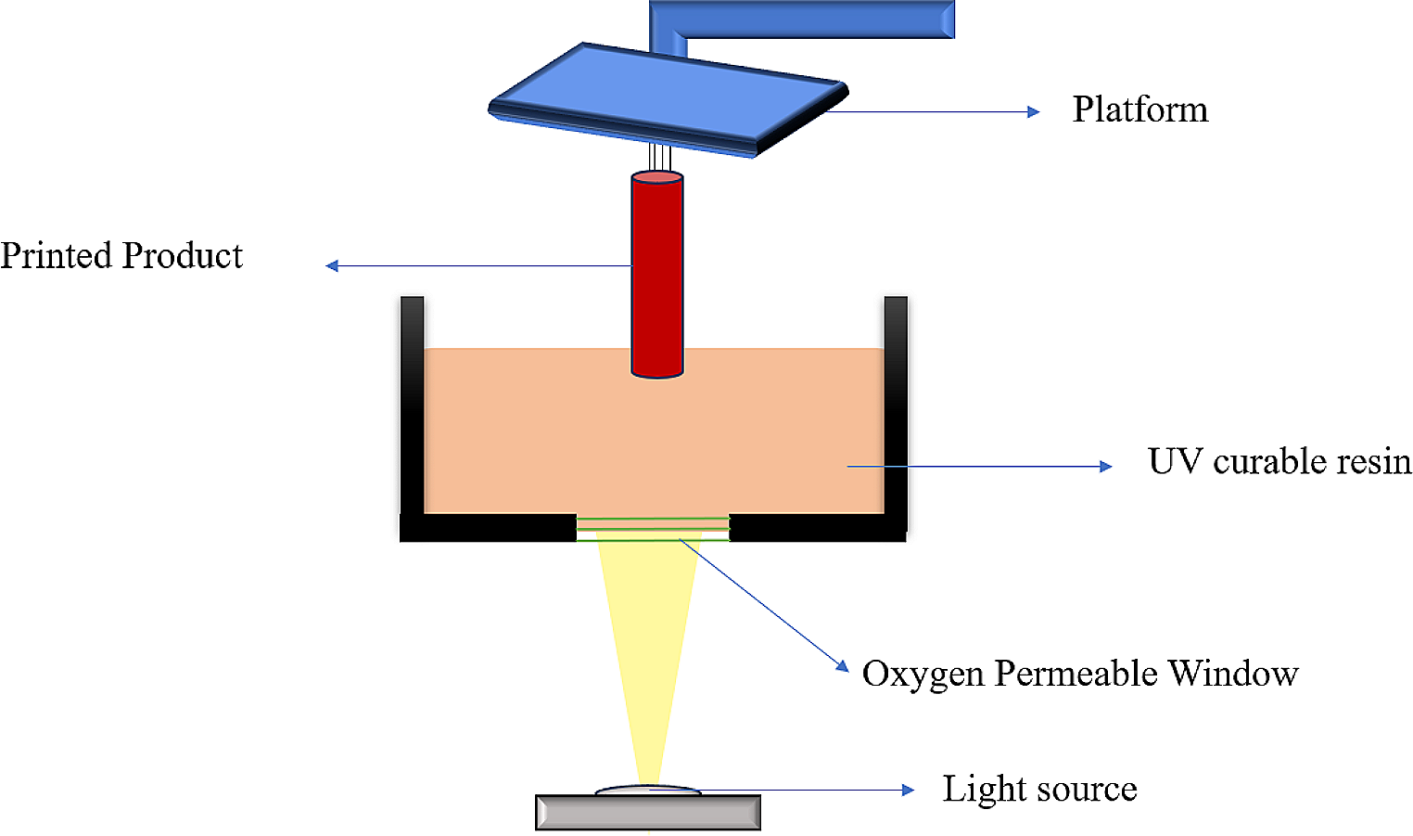
Schematic diagram of working of CLIP

CLIP is a more advanced and rapid method of 3D printing technology than SLA. The CLIP system focuses light onto the photopolymerizable material made of individual pixels from whole cross-sectional layers of objects. The illustration of the instrument is shown in Fig. 7. Compared to the digital projector screen used in DLP systems, CLIP employs higher-end digital projectors based on light-emitting diode (LED) or laser technology. Unlike SLA and DLP printers, which use layer-by-layer production, the oxygen-permeable layer in CLIP printers produces a dead zone above the Vat’s base. It allows for continuous product printing [37]. The 3D printer has a Vat filled with a liquid photopolymer resin. Part of the Vat bottom is transparent to UV light, acting as a “window.” A digital projector shines UV light through this window, selectively curing the resin in the desired pattern for each layer. An oxygen-permeable membrane below the resin creates a “dead zone” - a persistent liquid interface that prevents the resin from fully curing and sticking to the window. As the UV light cures the resin, the build platform slowly pulls the object out of the Vat, allowing uncured resin to flow under and maintain contact with the bottom of the object. This continuous, layerless printing process allows CLIP to create objects much faster than traditional layer-by-layer 3D printing techniques. The oxygen permeable layer comprises monomers prone to free radical photopolymerization, react with oxygen, and produce peroxy radicals, thus avoiding photopolymerization. The more stable peroxy radicals cannot easily restart the polymerization. Therefore, the production of peroxy radicals is prevented by making the resin flow constantly between the polymerized material and the Vat’s base, making it simple and easy to polymerize. When CLIP systems are compared with SLA and DLP, the former can print 25–100 times faster [38]. However, the main downside of CLIP systems is the need for a low-viscosity polymeric resin and an expensive oxygen-permeable layer [37].

Johnson et al. developed a single-step MN array fabrication process with customizable geometries to manufacture the patch (time taken less than 10 min per patch) using the CLIP technique. The study involved the rapid fabrication of MNs with various sizes, shapes, aspect ratios, spacings, and formulas in minutes compared to hours. Among the variety of MNs, their findings suggested that CLIP 3D printing can be utilized for manufacturing, encapsulating, and controlling the release of therapeutics from square pyramidal MNs with a variety of compositions, including polyacrylic acid, polyethylene glycol, and photopolymerizable derivatives of polycaprolactone and trimethylolpropane tri acrylate [39].

These are the techniques used to fabricate the MNs. Various 3D printing techniques utilized by the researchers are summarised in Table 1, along with their advantages and disadvantages.

**Table 1:** Different types of 3D printing techniques with advantages and disadvantages

Method of manufacturing of 3D printed MNs	API and materials	Description/ Significance of work	Advantages	Disadvantages	References
FDM	Fluorescein dye, polylactic acid (PLA)	Rapid printing with tailored needle density, length, and shape of MN was achieved by FDM. PLA swellability was utilized to load small-molecule drugs.	Inexpensive, simple fabrication technique, easy to fabricate, high speed.	Low speed, drug degradation is expected, and lack of accuracy.	[27]
FDM	Estradiol valerate, PLA	Using FDM 3D printing, biodegradable polymer-based MNs were created for estradiol valerate’s long-term, painless transdermal administration. A PLA filament drug reservoir was produced with an FDM 3D printer (Quantum 2025 desktop printer, Persia 3D printer Co., Tehran, Iran). The injection volume filling method was used to fill the drug reservoir of the MN arrays.	This three-dimensional (3D) printing technique is inexpensive and quick, enabling the accurate and quick manufacture of MNs.	Limited drug loading capacity, the possibility of thermosensitive pharmaceuticals degrading during the printing process, and constrained resolution for accurately producing the small needles of MN arrays.	[40]
TPP	Silicone	The MNs were found to be stable within the skin tissue.	High-resolution, various photo-resist substances are used to create multifunctional micro/nanostructures.	Lack of proper information on how photo-resistant materials affect the depth of micro/nanostructure.	[28]
TPP	Indium tin oxide coated Polyethylene terephthalate film.	A novel method for creating hollow MNs was developed by combining the direct laser writing technique and TPP with optimized manufacturing settings to create microstructures precisely and quickly in three dimensions.	The capacity to create hollow 3D microstructure devices precisely, quickly, and adaptably with tiny openings and spatiotemporally regulated TPP reactions opens the prospect of producing customized hollow 3D microstructures with nanoscale precision. They produce structures with sizes, distributions, and designs that the user specifies.		[41]
TPP	2-Hydroxy-4′-(2-hydroxyethoxy)-2-methylpropiophenone and ormocer photoresin	A novel bioinspired design for MNs was created to administer medication or a vaccine. In the new design, the external scent efferent systems of some European true bugs inspire the structures adorning the lateral surfaces of pyramidal MNs. These structures allow for directed liquid movement. The TPP 3D printing method was employed to create these tiny needles.	Generating structures of user-defined designs, sizes, and distributions		[42]
SLA	Cisplatin	Specialized cross-shaped MNs demonstrated potent anticancer efficaciousness, tumor suppression, and exceptional skin penetrating capacity.	It produces parts with a realistic finish and complex shapes with the best resolution, accuracy, and surface finish.	It is more expensive than other 3D printing methods. It has a limited build volume, so it can’t create large objects. SLA uses a liquid resin, which can be messy.	[32]
SLA	Rifampicin	Biocompatible SLA resin was used to create hollow MNs that could potentially used to deliver high molecular antibiotics, such as rifampicin, which have hepatotoxicity, limited bioavailability, and stomach instability. To achieve great mechanical strength and minimal tissue occlusion with a significant storage capacity, holes were formed at the tip of a hollow MN.			[43]
DLP	Gold/silver nanoclusters	DLP was used to construct a polyvinyl alcohol (PVA) based MN patch effectively. Once the patch was readily peeled off the skin, nanoclusters were released from the patches.	Good grade material, smooth surface, high accuracy and precision, and better suited for hollow MNs	Poor bioavailability expensive, weak mechanical strength	[36]
DLP	Vitex agnus-castus and Tamarindus indica extract with polyvinyl pyrrolidone.	Using the DLP technology, polymeric MNs containing extracts from Tamarindus indica and Vitex agnus-castus were created to investigate the impact of the extracts on cellulitis and offer a new method for managing the condition.			[44]
High Precession DLP(HPDLP)	Polyethylene glycol diacrylate (PEGDA)	Polymeric MNs were created using the HPDLP technique. The impact of printing parameters on the MNs’ mechanical strength was also studied. Simulation experiments involving the puncture of MNs on artificial skin, as well as simulations involving drug injection and drug extraction, were carried out.	When compared to template-driven fabrication, the cost of the MN production platform is significantly lower with the accuracy and precision of the HPDLP system		[45]
CLIP	Polyacrylic acid, polycaprolactone, and polyethylene glycol MNs with fluorescein and rhodamine dyes	It was possible to create square pyramidal MNs with different kinds of polymers. The MN patch successfully released the fluorescent drug surrogate when inserted into mouse skin.	Fastest technique, high precision, and accuracy.	High-cost technique, Minimum mechanical property, appropriate only for oxygen-sensitive materials.	[39]

The most preferred 3D printing technique is FDM, among the other methods. FDM is the most common type of 3D printing due to various advantages, such as affordability. FDM 3D printers are generally more affordable than other 3D printing technologies like SLA or selective laser sintering (SLS). This makes FDM printers accessible to many users, including small start-ups. Second is the ease of use. This printing has a relatively low learning curve and is user-friendly, making it a popular choice for non-technical users. This makes it user-friendly. The printing process is straightforward, extruding the material through a heated nozzle. FDM can print with various thermoplastic materials, including PLA, Acrylonitrile butadiene styrene (ABS), Polyethylene terephthalate glycol (PETG), and specialty filaments like wood, metal, and carbon fiber (CF) composites. This material versatility allows users to create diverse 3D-printed objects. Thus, the combination of affordability, ease of use, material variety, part strength, and accessibility make FDM the most preferred 3D printing technique for a wide range of applications [46].

The second method chosen is photopolymerization-based techniques in TDD. Among all the techniques, photopolymerization-based technologies SLA and DLP offer variability in the final product’s optical, chemical, and mechanical properties since the polymer chemistry could be versatile. This technique is based on monomers/oligomers that can be photopolymerized in the presence of a photoinitiator upon light emission to a reservoir (vat) filled with photocurable liquid resin. The light source is versatile according to the utilized photoinitiator system, and the polymerization process is either radical or cationic. Mainly, SLA printing has been widely applied to overcome several drawbacks of conventional transdermal MNs, such as inadequate sharpness, imprecise control of drug release, and insufficient drug permeation through the skin, which demonstrated great potential in the fabrication of personalized MNs with superior drug delivery efficiency [47]. The techniques can be chosen as per the requirement and applicability.

## Polymers used in 3D printing

The main element of the 3D printing process is the material used. The resin (ink) comprises photosensitive monomers/oligomers capable of undergoing photopolymerization on exposure to light to form polymers. Therefore, photopolymers are of utmost importance and vital components in 3D printing; recent times have witnessed many advancements that have led to the development of various biocompatible photopolymers that are suitable for 3D printing technology. Some of the examples are PEGDA, polyethylene glycol dimethacrylate (PEGDMA), poly (2-hydroxyethyl methacrylate) (pHEMA), and polypropylene fumarate (PPF). The choice of materials is pivotal in determining the 3D printing fabrication technique. Due to its heat-dependent deposition mechanism, FDM predominantly employs thermoplastic polymers as its feedstock. While PLA is favored for its relatively modest melting point, biocompatibility, and degradability through hydrolysis, PVA is also a popular option due to its excellent biocompatibility, robust mechanical strength when dry, capacity to transport solute in a gel state post-skin insertion, short-term gel integrity retention, and eventual dissolution and absorption into the skin. In the 3D printing industry, polymeric materials in a liquid state and those with low melting points are favored due to their affordability, lightweight nature, processing versatility, and ability to offer mechanical support. Despite this, there has been a growing adoption of materials with higher melting points, such as polyether ether ketone (PEEK) and polymethyl methacrylate (PMMA). On the other hand, the starting materials for vat photopolymerization fabrication, which depends on light-mediated crosslinking, typically comprise photoreactive monomers or oligomers and photo-initiators. Several studies have been conducted on the formulation of biocompatible polymers for vat photopolymerization fabrication, allowing for the possibility of incorporating different properties into the printed parts [21, 30, 48–50]. Here, we summarise some commonly used polymers in the 3D printing process.

### Thermoplastic polyurethane (TPU)

TPU is a type of elastomer under thermoplastic elastomers (TPE). TPU is formed by the reaction between Polyols (polyester, polyether, or polycarbonates(PC)-based;), diisocyanates, and short-chain diols. This thermoplastic elastomer can be melted and is very flexible, strong, and resistant to abrasion. TPU is a compound comprising block copolymers with linear segments that have soft and hard portions. A polyol typically comprises the soft portion, and a diisocyanate and chain extender comprise the hard portion. The rigid portion of the polymer provides rigidity to its initial shape, and the soft portion reserves energy for dissipation to return to its original form when exposed to external stimuli. TPU has a melting point of 235℃ and Young’s modulus ranging from 5 to 100 MPa. Depending upon the physiochemical property of the polymer, a suitable 3D printing technique is used for processing the material [51].

### ABS

ABS is one of the first and most extensively utilized polymers in 3D printing. It is a petrochemical triblock copolymer created using polybutadiene and has high strength and flexibility. It is a high-impact engineering thermoplastic and amorphous polymer. ABS comprises three monomers: acrylonitrile, butadiene, and styrene. It exhibits strong chemical resistance against chemicals and can be used in situations that involve contact between various substances, like chemical processing and laboratory environments. ABS polymer has very low shrinkage during 3D printing, so there are fewer chances of warping or distortion while printing complex structures. As a result, print quality overall and dimensional accuracy are improved. Because ABS is not biodegradable, it may have limited applications, and the polymer needs to be processed by the body to be eliminated or completely removed. This may result in a build-up of the polymer within the body, potentially leading to adverse effects. ABS can easily withstand temperatures between 20 to 80ºC and has a melting temperature of 105ºC. ABS is an attractive candidate for FDM and SLA 3D printing systems [52].

### PCs

PCs belong to the class of thermoplastic polymers, comprising carbonate groups in their chemical composition. PCs are biomaterials with exceptional durability that can withstand physical deformation up to 150℃. However, PCs are prone to absorbing moisture from the surrounding environment, which may impact printing resistance and performance. PCs show significant interactions with specific polymers like ABS Polyethylene terephthalate (PET), which are (PC)/ABS, PC/PET, and PC/ PMMA, PC/PET blends. Most amorphous polymers become hard and brittle below their glass transition temperature, whereas PC maintain their ductility. Hydrophilicity is one of the most important features of PCs, and it is suitable for biological applications such as drug delivery systems and polymeric scaffolds for tissue regeneration [53].

### High-performance polymers, PEEK

PEEK is an organic thermoplastic colorless polymer. PEEK is not often used in 3D printing because of the shortage of appropriate stock, requires high melting temperature, concerns about poor layer adhesion, and expensive processing procedures that are costly and labor intensive. PEKK biomaterials outperform many thermoplastic composites in terms of mechanical durability, chemical resistance, and thermal stability. The features of PEEK, such as radiolucency, minimal moisture absorption, and biocompatibility, make it an excellent material for use in craniofacial and bone tissue engineering [54].

### Polypropylene

Polypropylene is a crystalline thermoplastic polymer of propylene (propene) monomer. It is one of the lightest polymers among all standard plastics. It is well-known for its rigidity, shock absorption capacity, and resistance to abrasion. It is an affordable polymer with many characteristics, including dimensional stability, flame resistance, transparency, and recyclable nature. Limitations associated with the polymer are low-temperature tolerance (brittles below 20 °C), susceptibility to UV radiation that can cause it to expand, and quick swelling in aromatics and chlorinated solvents. Because of its very lightweight and rigid characteristics. It is frequently utilized to create customized 3D-printed orthoses for patients with bone fractures. It has anti-thrombogenic properties that may be improved to support plasma protein adsorption, adhesion, and platelet activation, which are critical for biomedical applications [55].

### PLA

PLA is one of the most commonly and widely used polymers in FDM 3D printing due to its affordability, quickly processed, biocompatible and biodegradable. Although its high cooling and solidification speed makes it challenging to work with, it can be easily extruded between 190℃ and 230℃ (melting temperature 175 °C). Because PLA has comparable compressive strength to bone and strong mechanical qualities, it is mainly used in musculoskeletal tissue engineering. Further, PLA can be used with ceramic to enhance the mineralization and compressive strength after implantation. One of the main issues with PLA is its long-term biocompatibility due to the generation of lactic acid byproducts during breakdown. This might result in tissue irritation (in the case of orthotics) or cell death. To address this problem, PLA polymers are combined with carbonated calcium phosphates, which may neutralize acidity and buffer within the physiological pH [56].

### Photocurable resins in vat photopolymerization

Based on the reaction mechanism, photocurable resins may be divided into ionic and free radical categories. Because of their rapid reaction rates and adjustable mechanical characteristics, Methacrylate and acrylate-based monomers are frequently used in free radical reactions. Epoxy resins are popular cationically polymerized photopolymers cured via step-growth polymerization in the presence of amines or anhydrides. Epoxies are commonly preferred over acrylates because of their better mechanical qualities and low shrinkage behavior, which results in greater dimensional accuracy during fabrication, although they take longer to cure. The critical drawbacks of photocurable resins are their low recyclability, biocompatibility, inability to print high-viscosity resins, and the necessity of printing with support structures. A mix of epoxy and acrylic resins is needed to benefit from the best qualities of both systems. After completion of the reaction and cross-linking, the systems form an Interpenetrating polymer network (IPN), and the characteristics may be customized by varying the mixing ratio [18, 57–59].

### Polymer composites

Polymer materials with low melting points or in liquid form are commonly employed in 3D printing because of their low weight, affordability, and processing flexibility. The lack of mechanical strength and functionality of 3D printed polymer products is challenging for their wide applications. Using different substances in combination with polymer to achieve desired mechanical and functional qualities is an effective remedy to these issues. As a result, there has been much interest in creating composite materials compatible with 3D printers, which has intrigued tremendous attention in the market. Numerous advances in developing novel printable composite materials reinforced by fibers and particles have been proven.

#### Particle-reinforced polymer composites

Particle reinforcements are frequently employed to enhance the polymer matrix’s characteristics. Combining particles with polymers, regardless of liquid or powder form, is simple for SLA or SLS or extruding printable filaments for the FDM process. The primary considerations to take into account when 3D printing particle-reinforced composites are improved tensile/storage modulus by adding glass beads, iron or copper particles, improved wear resistance by adding aluminum and aluminum oxide (Al_2_O_3_), and improved dielectric permittivity by adding ceramic or tungsten particle. The incorporation of particles aids in resolving some printing-related issues. One of the main issues with FDM 3D printing is the distortion of final printed parts due to the thermal expansion of the polymer. It has been demonstrated that integrating metal particles into polymers effectively addresses this issue.

#### Fiber-reinforced polymer composites

Fibre reinforcements can significantly improve the characteristics of polymer matrix materials. Fiber-reinforced polymer composites are often produced using FDM and direct write methods in 3D printing. Polymer pellets and fibers are combined in a blender before they are taken to an extruder to be made into filaments for FDM processing. A second extrusion process can be performed to ensure that fibers are uniformly distributed. Fibers and polymer paste were mixed before and then extruded for direct writing processing. Usually, short fibers such as CFs and glass fibers are often employed for reinforcements to enhance the mechanical characteristics of polymer composites in 3D printing. Fiber orientation and void fraction determine the features of the final composite parts. ABS/CF composites made by FDM exhibited increasing tensile strength and modulus with maximum increases of 115% and 700%, with the fiber percentage increasing at 40 weight% fiber loading, as shown by Tekinalp et al. Ning et al. investigated the effect of fiber content on the mechanical characteristics of ABS/CF composites manufactured using FDM. The most significant results were seen at five weight% fiber loading, and good printed products were obtained. In contrast, higher fiber loading negatively impacted the performance of the printed part because of increased porosity [60].

## Applications of 3D printed MNs

The use of 3-dimensional techniques has several distinct advantages. 3D printed materials can include a diverse solution to the controlled release of the drugs, minimum invasive delivery, high precision tracking, biomimetic models for drug development and manufacturing, and a future chance for personalized medicine at the micron level. Biomimetic MNs can replicate natural structures like plant thorns or insect stingers, which are used to penetrate the skin to deliver drugs, peptides, etc. The manufacturing process is different than regular 3D printing and is called bioprinting [61]. It involves mimicking of biological processes in a controlled environment like angiogenesis, tumorigenesis etc [62]. One of the most groundbreaking and successful methods for modifying and personalizing medication formulations is using 3D printers. Here are a few examples of how the 3D printing technique has been used to enhance the drug distribution method and overcome barriers.

### Treatment and drug delivery

#### Transdermal delivery of anti-wrinkle small peptide through tailored 3D printed MNs

Wrinkles appear as curves or creases in the skin and are the earliest symptom of aging. The periorbital zone is one of the most wrinkle-prone areas on the face. Successful wrinkle care is needed to enhance these people’s standard of living and self-care. Botox was once considered among the most common and efficient wrinkle-reduction procedures. However, because of its toxicity and other side effects, it has been tightly controlled. Acetyl hexapeptide-3 (AHP-3), or Argireline, is a topical Botox-like product that blocks the release of Acetylcholine neurotransmitters and decreases the repetitive constriction of internal muscles that control facial speech, reducing hyperkinetic wrinkles and facial lines. Because of its low risk and non-invasiveness, this can treat wrinkles instead of Botox, which would be inserted straight into the facial muscle. AHP-3 has also been discovered to be an efficient antiwrinkle product, with findings showing a 49% increase following four weeks of twice-daily therapy. However, because of its high molecular weight and reduced LogP value, AHP-3 has poor penetrability across the skin [63].

The authors concluded that photocurable resins available for 3D printing are inappropriate for manufacturing drug-loaded delivery methods. Two liquid biocompatible monomers, vinyl pyrrolidone (VP), and (PEGDA), were examined as photocurable resins for fabricating MNs. Their key characteristics, such as the final polymer’s physical strength, polymerization rate, swelling rate, 3D printing resolution, and safety profile, were assessed in different proportions. The optimal ratio of resin, corresponding to the parameters, was 7 VP: 3 PEGDA in weight. The introduction of AHP-3 to the ideal resin remained intact with the polymer throughout the fabrication process and had little influence on the physical properties of the final polymer. A customized MN patch was created utilizing computer-aided design software and then printed using optimized resin on a DLP 3D printer using a 3D scanned face model. The ability of a manufactured MN patch to penetrate and remain intact after compression was demonstrated in an in vitro analysis on human cadaver dermatomed skin. As a result, a photopolymer-fabricated personalized MN patch may be a novel way to transmit AHP-3 to the skin for successful wrinkle treatment [63].

#### Mesoporous iron oxide nanoraspberry with PVA MN for androgenetic alopecia treatment

Male baldness is a genetic disorder that often affects males between 30 and 60. It is estimated that over 50% of people suffer from hair loss. In earlier times, the method of treating genetic male baldness was by blending active ingredients with inert substances like propylene glycol and glycerol, which would help soften the stratum corneum and facilitate the absorption of active drugs. It takes a while for the stratum corneum to soften, and some users have reported adverse effects like rash, redness, swelling, and subjective burning sensations. Additionally, incorrect techniques might result in hair growth in the undesirable region. MNs are a novel method of painless administration that quickly penetrates the stratum corneum, which allows drugs to reach the epidermal and dermal layers while being easy to use, minimally invasive, and reasonably affordable [64].

Applying DLP 3D printing technology, Fang et al. and coworkers created a magneto-responsive composite MN containing a mesoporous iron oxide nano raspberry (MIO) to be given transdermally to increase the drug delivery efficiency. The rationale for using DLP 3D printing technology was that it could produce MNs of different sizes, shapes, and widths. In addition, it offered advantages over traditional processes, such as high resolution, low cost, flexible manufacturing, customization, and quick prototype formation. For hair regeneration, a therapeutic drug called minoxidil was encapsulated in the composite. When the external magnetic field is applied, the local temperature rises, and the drug is released in a controlled manner. Additionally, the mechanical characteristics and biocompatibility of MNs were examined [64].

#### 3D printed MN for delivery of insulin

MN systems have received a lot of interest since they were first developed because they have the potential to replace conventional medication delivery methods. Most patients with type 1 and some varieties of type 2 diabetes rely on subcutaneous needle injections to replenish their insulin; nevertheless, this method of therapy is strongly linked to worse patient compliance. Much research has been done on MN-based devices for transdermal insulin administration due to factors including pain, skin thickening from repeated injections, needle fear, and insulin leakages on the skin’s surface. Recent developments include the utilization of moulding processes to create systems for dissolving MNs that are loaded with insulin.

Using a commercial SLA printer and a biocompatible Class-1 polymer, 3D-printed MN arrays with the spear and pyramid MN designs were created. Then, employing inkjet printing, insulin-sugar films were applied to the 3D-produced arrays. Inkjet printing is used for modification of the surface of the MN by uniformly coating biologically relevant materials. It generates patterns with various geometry. It could overcome the disadvantages caused by dip coating methods like dipping, withdrawal, and drying cycles [65]. Mannitol, trehalose, and xylitol were used as insulin carriers to maintain insulin activity before active films were deposited on the surface of the MNs. In vivo and in vitro studies showed that coated MN systems released insulin quickly [66].

#### 3D printed MNs for electronically controlled transdural drug release

After a spinal cord injury (SCI), inflammation and cytotoxicity can cause further damage to neural cells, with secondary injury mechanisms like neuroinflammation, excitotoxicity, and ischemia contributing to ongoing damage. Excitotoxicity, due to excess neurotransmitters, free radicals, and reactive oxygen species, leads to cell death around the injury site. Free radicals, such as nitric oxide released by inflammatory microglia, can damage nearby uninjured tissues, resulting in secondary neuron death for weeks post-injury. The researchers formulated a conductive, biocompatible polymer, polypyrrole (PPy), for electronically controlled drug release via implanted electrodes to address this issue. A PPy MN array was fabricated for transdural drug delivery, allowing local delivery of neuroprotective or anti-inflammatory drugs like dexamethasone phosphate (Dexa), known to reduce neuroinflammation and apoptosis. The MNs were created using microscale 3D printing with TPP, UV curing, and coated with chrome, gold, and PPy. In vitro studies showed that Dexa PPy MNs significantly decreased NO, IL-6, and MCP-1 in a microglial model of neuroinflammation without causing cell death. In vivo, the PPy MNs effectively delivered (Dexa) through the rat dura mater, demonstrating the potential for epidural drug delivery to the spinal cord [67].

### Fluid extraction and sampling

#### 3D printed MNs in interstitial fluid extraction (ISF)

The innovation of MNs has been recognized as a novel approach to medication delivery and ISF extraction. MNs of different lengths (1000 μm, 1500 μm, and 2000 μm) were selected, and their ability to extract ISF was determined [68]. Fluid was extracted from all three lengths of MNs, and a needle with a length of 1500 μm collected the highest percentage of ISF with a total volume of 1.51 µl from the single needle, compared to other lengths. Arrays of MNs were then developed to enhance quantities of ISF extraction volume. The MN array, having an open concentric shape at the base, was constructed by 3D printing, allowing each array to hold up to five needles in parallel. Using these arrays, a total volume of 16 µl was extracted from human volunteers at 1–2 h intervals. MNs of varying shapes were developed, having flat, concave, convex, or bevel profiles with distinct extraction efficiencies and penetration depths up to 1500 μm. According to the authors, the MNs holder’s configuration parameters impact ISF extraction, and a concave tip shape is best for extracting ISF from animals [68, 69]. The design of MNs plays a crucial role in effectively extracting ISF from animals. The particular parametric studies have shown that the shape of the MN tip is an essential factor, with concave tip profiles demonstrating significantly improved ISF extraction rates compared to flat, convex, or beveled tips in animal models like CD hairless rats. MN holders with concave tips could extract 0.85 ± 0.64 µL/min of ISF, which was optimal for this application [68]. The length of the MNs is another critical design consideration. In a pilot study on human subjects, 1500 μm long MNs had the highest success rate of 31% for extracting ISF, outperforming 1000 μm (14%) and 2000 μm (16%) lengths. The average total ISF volume extracted per MN was 1.51 µL, highlighting the importance of selecting the appropriate needle length [70]. Beyond tip shape and length, other MN design parameters, such as tip angle and width, can also influence ISF extraction. Sharper tip angles and narrower widths generally allow for easier skin penetration and lower insertion forces, which can enhance ISF flow. The choice of MN material and fabrication method is also crucial, as these factors impact properties like strength, sharpness, and hydrophobicity, all of which affect insertion and ISF collection [68, 71].

#### Sampling of perilymph from guinea pigs for proteomic analysis

Diagnosing the inner ear is limited due to the inability to extract the inner ear fluid. Therefore, a round window membrane (RWM) is the portal to access the perilymph samples. The researchers used 3D-printed hollow MNs of 100 μm outer diameter and 35 μm inner diameter. The perilymph samples (1 µL) analyzed by liquid chromatography-mass spectrometry-based quantitative proteomics showed hollow surgical MNs safely facilitated the aspiration of 1 µL of perilymph from the guinea pig scala tympani through the RWM. The perforation of the RWM with a 100 μm diameter MN did not cause lasting anatomic or functional damage. In animals, proteomic analysis of the inner ear thus far has required the harvesting of cochlear tissue involving sacrifice of the animals or sampling of perilymph using traumatic methods of cochleostomy for perilymph sampling and dissecting the cochlea from the skull, removing up to 10 µL of perilymph with a syringe. Using 2PP 3D-printed MNs represents the first non-traumatic perilymph sampling method successfully used in survival experiments to facilitate perilymph analyses. Furthermore, the absence of cochlear damage and complete RWM perforation closure makes repeated perilymph sampling possible should it be necessary [72].

### Innovative design

#### Limpet tooth-inspired 3D printed MNs

While 3D printing technology is rapidly improving the manufacturing process of MNs, a significant issue remains with the inadequate mechanical performance of each needle. Some animal tissues have unique microstructures, and their biomaterials have demonstrated remarkable mechanical properties. It has been shown that the teeth of limpets, aquatic snails with shells that stick to rocks, may be the most challenging component in the natural world. Microscopic limpet teeth have extraordinarily high mechanical strength because of their unique arrangements of several goethite nanofibers parallel to the surface. 3D printing has generated more options for modifying and creating multi-scale, multi-material, flexible, adaptable structures with improved mechanical performance [73].

Li et al. and colleagues developed and created limpet teeth-based 3D printed MNs with reinforced iron oxide particle (IO)-based nanofillers. MNs were made using a magnetic field-aided 3D printed technique. Aligned Iron oxide nanoparticles (aIOs) were suspended within a polymer matrix and incorporated into MNs for painless administration. The manufacturing approach used magnetic control for perfect micro-filler alignment, selective photocurable polymer crosslinking, and magnetic field-3D printing to enclose the aligned nanoparticle bundles within the printed MNs. MNs of limpet teeth containing aligned iron oxides (aIOs) and random iron oxide nanoparticles (rIOs) were compared. The former showed better mechanical properties. Limpet teeth conceptual MNs showed enhanced mechanical strength due to depositing iron oxides layer by layer, thus increasing effectiveness. MNs made with the magnetic field-aided 3D Printing (MF-3DP) process have overcome the constraints of those made with traditional production procedures. The MF-3DP process helped to reduce patient pain while administering vaccines or hypodermic needles. MNs developed using MF-3DP are small and possess sharp tips, so the insertion force required during administration is less, and the potential contact area with pain-sensing neurons is less. Due to the exact deposition depth, 3D-printed MNs may offer an opportunity to govern how the vaccine delivery is carried out to the epidermal and dermal layers of the skin, which is difficult to achieve with hypodermic needles. According to the overall in vivo results, the study observed that mice did not exhibit any noticeable reactions to the presence of the MNs. The MNs remained firmly attached to the skin and enabled comfortable wear during prolonged periods, showcasing their potential for painless drug delivery applications. It was confirmed that MF-3D printed MN array can deliver medications to humans and animals for an extended period [73].

#### 3D printed dissolving MN arrays for vaccination

MNs target the skin in the form of in situ cutaneous injections. In one study, authors made novel dissolving undercut MNs as an immunization strategy. They have used 3D-laser lithography to formulate the MN with unique geometries and micro-moulding with desirable materials. Dissolving MN arrays, wherein the tips were loaded with multiple cargos and ovalbumin antigen was loaded along with the adjuvant. This MN design consisted of a sharp-tipped pyramid head and an undercut stem portion with a filleted base. The MN was 750 μm in height with a 30° apex angle. The final dosage form could take multiple cargos and be delivered to murine and human skins. The formulated vaccine could produce a sustained immunogenic effect through antigen-loaded MNAs. It made more potent antigen-specific cellular and humoral immune responses than traditional immunization by intramuscular injection [74].

## Recent patents on 3D-printed MNs

Several patents have been filed in the area of 3D-printed MNs. Some of them are listed in Table 2.

**Table 2:** Patents on 3D-printed MNs

Sr.no.	Patent No	Title	Description	References
1	US11213667B2	3D printed MNs for microencapsulated mammalian cell extrusion	A 3D-printed biocompatible drug delivery device is provided, with a fluid delivery channel distinguishing three segments and a receiving chamber with an array of MNs.	[75]
2	CN110693855B	A preparation method and application of a 3D printing MN patch	The preparation method of MNs using FDM and the application of a minimally invasive 3D printing MN array patch capable of intelligently releasing insulin and adjusting blood sugar.	[76]
3	US11697008B2	A method for fabrication of 3D printed MN assembly	A 3D-printed MN array was created by exposing a photo-polymer liquid resin to a light source layered through a series of patterns projected onto the liquid resin.	[77]
4	US11628208B2	System and method for MN delivery of microencapsulated vaccine and bioactive proteins	Microparticles or nanoparticles of the encapsulated vaccine are produced by preparing the vaccine antigen and biocompatible polymer solution and spray drying the solution to form encapsulated microparticles or nanoparticles. Further, microparticles and film polymer solution suspension are formed and loaded into the 3D printer to obtain the vaccine-loaded MNs.	[78]
5	CN112370649	They are individually customized MN beautifying face masks and preparation methods.	The characteristics information is obtained using 3D scanning technology and 3D printing technology to fabricate the upper mould and lower mould according to the personal face characteristics. Further laser technology is used to carry out targeted etching of the MN holes on the upper mould through a hot press moulding technology. The individual MN face mask, which conforms to the face characteristics of each person, is manufactured.	[79]

## Conclusion with a future view

The various favorable features of MNs, such as self-administration, painless drug delivery, and minimum invasion, make MNs one of the emerging fields for future TDD. The diverse types of MNs, such as hollow, coated, and solid, enable various applications. 3D printing has revolutionized conventional fabrication processes in several areas, mainly health care and pharmaceutics, taking patient-specific treatment one step closer to existing ones. The addition of 3D printing technology makes MNs more potent and attractive for various additional applications with personalized drug administration. Different methods have been used for the 3D printing of MNs, such as SLA, DLP, and FDM, which have been developed continuously in recent years. This advanced technology enables design as per the need of specific features. Availability of several printing materials such as different types of epoxy resins, aryl acid esters, phosphoric acids, diacrylate, phosphine oxide, and polyethylene glycol all these materials play a crucial role in the fabrication of various types of 3D printed MNs. This advancement in 3D printing techniques ensures that 3D printing MNs overcome the drawbacks of conventional MNs.

Fabrication of 3D-printed MNs is a sophisticated technique that requires appropriate printing material, ideal printing parameters, and skilled operators with high knowledge. There is still a need to make 3D printing techniques easier and adaptable with high accessibility and low cost. The use of 3D printed MNs in real life for their practical clinical application is not yet being done; conventional MNs are being used for large-scale production as the 3D printing method is not being used at large-scale production. This provides the scope for future research using 3D printing techniques for large-scale manufacturing. Another challenge is to address the drawbacks of present 3D printing techniques [80].

## Data Availability

Not applicable.
